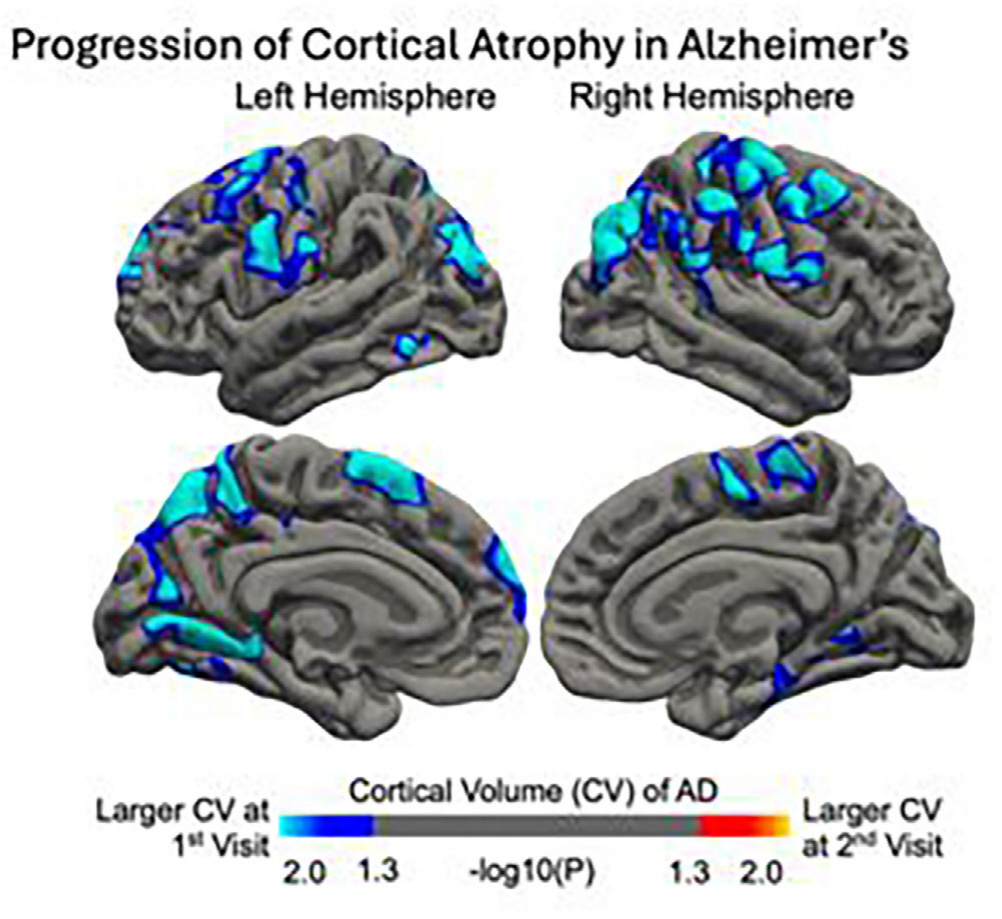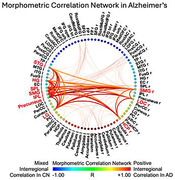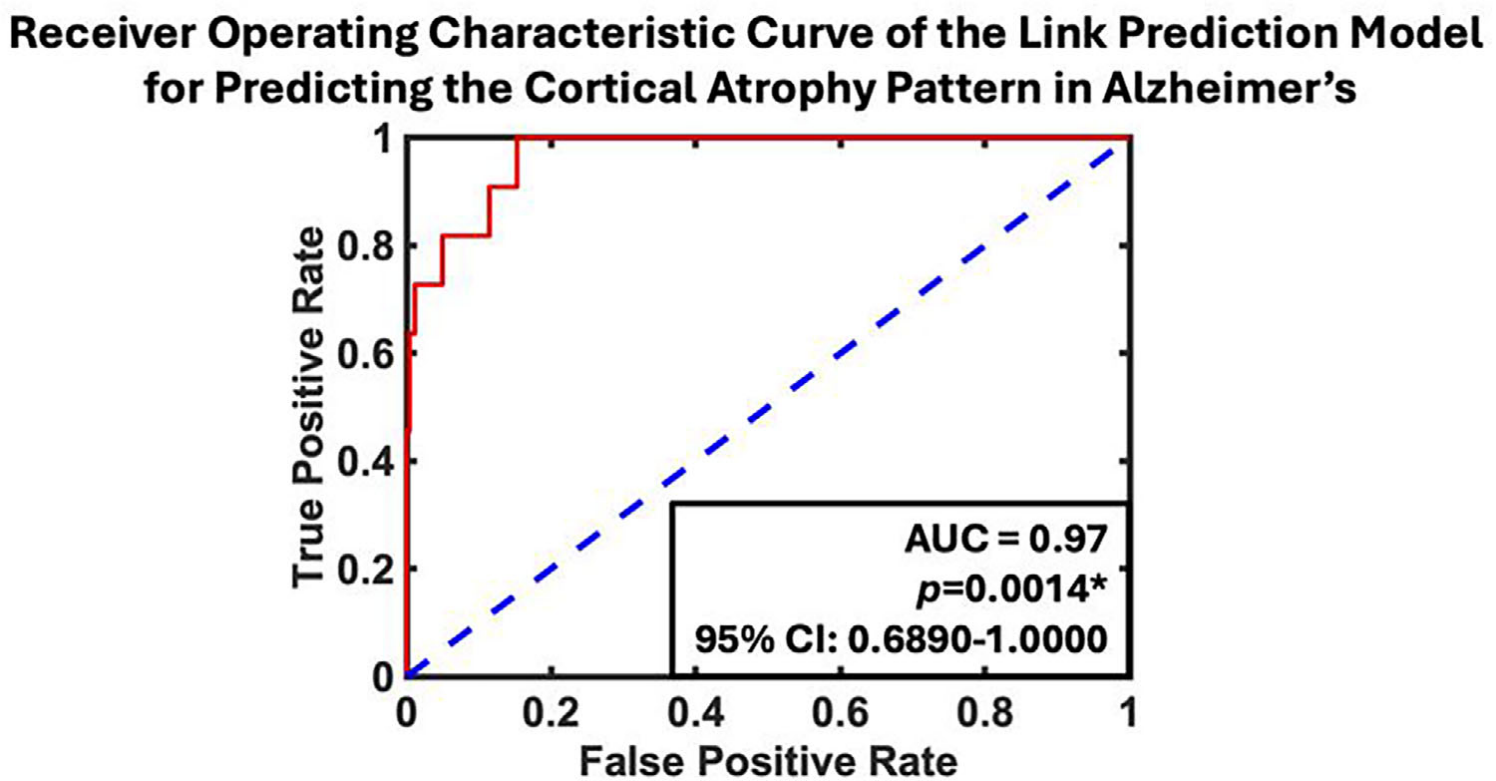# Link Prediction of Cortical Atrophy in Alzheimer’s Disease using Morphometric Correlation Networks

**DOI:** 10.1002/alz.094741

**Published:** 2025-01-09

**Authors:** Tiffany Luo, Chencai Wang, Benjamin M. Ellingson, Ceylan Z. Cankurtaran

**Affiliations:** ^1^ UCLA, Los Angeles, CA USA

## Abstract

**Background:**

Predictive biomarkers characterizing disease progression are called for in the context of emerging treatments for Alzheimer’s disease. We implemented a link prediction model on morphometric correlation networks(MCN) generated from structural MRI.

**Method:**

High‐resolution T1MPRAGE images were retrospectively collected at two timepoints (interval 2.6 ± 1.2 years) for each of the 32 AD patients (AD)(mean age 74.6 ± 6.8, 14F/18M) and age‐matched cognitively normal controls (CN)(n = 32, mean age 75.0 ± 6.3, 16F/16M) from Alzheimer’s Disease Neuroimaging Initiative database. Images were processed using Freesurfer(version7.2) cortical reconstruction pipeline followed by cortical volume quantification. Whole‐brain surface‐based analyses (set at *p* < 0.05 and FDR < 0.05) were performed to identify regions showing significant differences between CN and AD, and significant atrophy between two timepoints across AD followed by computation of interregional correlations of cortical volume changes. Topological robustness of the MCNs for both timepoints were quantified to assess the network robustness of AD in response to the removal of predominant nodes. The MCN was used to build a link prediction model. Graph embedding was implemented to learn and extract features of each node in the MCN, and the light gradient boosting machine algorithm was applied to classify the cortical changes as atrophy. The AUC(area under the receiver operating characteristic curve) was used to evaluate the model’s performance.

**Result:**

AD showed widespread cortical volume reduction. When examining the longitudinal cortical atrophy, the left superior temporal gyrus (STGl,*p* = 0.0108), precuneus (*p* = 0.0262), inferior(IPLl, *p* = 0.0019) and superior parietal lobule (SPLl,*p* = 0.0007), lingual gyrus(LGl,*p* = 0.0004); the right lateral occipital cortex (LOCr,*p* = 0.0168) and hippocampus(HCP r,*p*<0.0001); and bilateral supramarginal gyri(SMGl,*p* = 0.0227; r,*p* = 0.0181) showed significant volume loss (**Fig. 1**) and were identified as predominant nodes in the MCN that led the co‐atrophy as AD progressed (**Fig. 2**). With AD progression, robustness was weaker and removal of predominant nodes significantly reduced network resilience. The link prediction model predicted the cortical atrophy pattern in AD with AUC of 0.97 (**Fig. 3**).

**Conclusion:**

We successfully implemented a model to predict progression of cortical atrophy and identified atrophy‐leading brain regions in AD. Future clinical translation of our approach can serve as an imaging biomarker.